# Cattle Target Segmentation Method in Multi-Scenes Using Improved DeepLabV3+ Method

**DOI:** 10.3390/ani13152521

**Published:** 2023-08-04

**Authors:** Tao Feng, Yangyang Guo, Xiaoping Huang, Yongliang Qiao

**Affiliations:** 1 School of Internet, Anhui University, Hefei 230039, China; ft2953487927@163.com (T.F.); guoyangyang_gyy@163.com (Y.G.); hxping@mail.ustc.edu.cn (X.H.); 2 National Engineering Research Center for Agro-Ecological Big Data Analysis & Application, Hefei 230039, China; 3 Australian Institute for Machine Learning (AIML), The University of Adelaide, Adelaide 5005, Australia

**Keywords:** cattle, segmentation, attention mechanisms, DeepLabV3+

## Abstract

**Simple Summary:**

Research on the target area segmentation of cattle can improve the precision management and breeding level of pastures. When the cattle target is divided in the scene, we can further analyze animal habits by combining the scene information (drinking area, feeding area, rest area, etc.), which is of great significance for guiding farming production and management. Therefore, in this study, an improved deep learning semantic segmentation algorithm was proposed to realize the segmentation of cattle regions, and its performance was also verified in an actual breeding environment. The cattle regions obtained in this study provide a data-driven and technical basis for further analyses of cattle habits and body conditions.

**Abstract:**

Obtaining animal regions and the relative position relationship of animals in the scene is conducive to further studying animal habits, which is of great significance for smart animal farming. However, the complex breeding environment still makes detection difficult. To address the problems of poor target segmentation effects and the weak generalization ability of existing semantic segmentation models in complex scenes, a semantic segmentation model based on an improved DeepLabV3+ network (Imp-DeepLabV3+) was proposed. Firstly, the backbone network of the DeepLabV3+ model was replaced by MobileNetV2 to enhance the feature extraction capability of the model. Then, the layer-by-layer feature fusion method was adopted in the Decoder stage to integrate high-level semantic feature information with low-level high-resolution feature information at multi-scale to achieve more precise up-sampling operation. Finally, the SENet module was further introduced into the network to enhance information interaction after feature fusion and improve the segmentation precision of the model under complex datasets. The experimental results demonstrate that the Imp-DeepLabV3+ model achieved a high pixel accuracy (*PA*) of 99.4%, a mean pixel accuracy (*MPA*) of 98.1%, and a mean intersection over union (*MIoU*) of 96.8%. Compared to the original DeepLabV3+ model, the segmentation performance of the improved model significantly improved. Moreover, the overall segmentation performance of the Imp-DeepLabV3+ model surpassed that of other commonly used semantic segmentation models, such as Fully Convolutional Networks (FCNs), Lite Reduced Atrous Spatial Pyramid Pooling (LR-ASPP), and U-Net. Therefore, this study can be applied to the field of scene segmentation and is conducive to further analyzing individual information and promoting the development of intelligent animal farming.

## 1. Introduction

With the advent of modern information technology, visual task research has increasingly focused on scene understanding and semantic understanding. Combining the interaction information between the target and its surrounding environment is more conducive to fully understanding the information in the scene, making it easier to understand and interpret the content in the overall image or video [1,2,3,4]. By classifying image pixels into specific categories, image segmentation can analyze the image globally, enhance its semantic understanding, and facilitate automatic detection, recognition, and localization. Traditional computer vision techniques mainly extract artificially designed features (colors, shapes, textures, etc.) from images or videos and combine them with machine learning algorithms to achieve recognition or detection. However, the overall accuracy heavily depends on the feature extraction method, and complex scenes, occlusion between livestock, and lighting will affect the extraction of features, thereby affecting the recognition effect. Compared with traditional computer vision methods, deep learning-based image segmentation technology can achieve higher precision, robustness, and efficiency in target segmentation, making it widely used in agriculture [5,6,7,8].

In smart animal farming, acquiring individual animal characteristics and body information is essential for pastures to accurately evaluate the health status, disease risk, and growth trajectory of each animal [9,10]. Traditional methods of obtaining such information rely heavily on manual measurement or recording, which are laborious and prone to measurement errors. Intelligent technologies such as sensors, computer vision, and deep learning tools are often employed to acquire individual animal information and enable real-time monitoring and analysis of animal behavior [11,12], health status [13], environmental adaptability [14] by providing effective management and decision-making support for pastures. With the increasing amount of attention being paid to food safety, more management requirements and responsibilities are being placed upon animal farms. The development and application of artificial intelligence technology has highlighted its efficient, real-time, and accurate characteristics in animal farm management, promoted the development of modern animal husbandry, and has also received widespread attention.

Among these artificial intelligence technologies, the use of deep learning image segmentation combined with large-scale feeding technology to obtain individual animal characteristics and body information accurately and efficiently has become a research hotspot in the field of animal farming. Based on the segmentation model, cattle target segmentation and contour extraction can be achieved, and the segmented images can be used to estimate the weight of cattle limping [15], respiratory rate detection [16], individual identification [17], body parameter [18], and weight estimation [19]. The accurate segmentation and extraction of cattle from their environment is crucial for obtaining information regarding individual characteristics and the body shape of cattle in an efficient manner. Researchers have utilized various deep learning-based segmentation techniques to achieve instance segmentation and the contour extraction of cattle targets with high accuracy. For instance, Qiao et al. [20] used the Mask R-CNN model to achieve a segmentation MPA of 92% and average distance error (ADE) of 33.56 pixels for cattle in complex feedlot environments, which outperformed the state-of-the-art SharpMask and DeepMask segmentation methods. Wu et al. [16] used the DeepLabV3+ network with ResNet101 as a backbone to achieve the target segmentation of naturally standing cows with a target segmentation accuracy and IoU of 99.4% and 98.7%, respectively, which can realize the accurate extraction of cow contour features. Deng et al. [21] proposed a semantic segmentation optimization method based on RGB-D for beef cattle images through using Fully Convolutional Networks (FCNs), which led to a 2.5% increase in PA, a 2.3% increase in CPA, and a 3.4% increase in MIoU in complex background scenarios. Animal target segmentation is a hot topic, and animal region segmentation is conducive to further research on the correlation between the target and other areas of the scene. However, the current research scenario is relatively singular, and the applicability and generalization of the model still needs to be verified further.

Furthermore, these approaches still face challenges regarding target segmentation and the identification of cattle under diverse and challenging conditions, such as multi-scenes environments and different breeds of cattle. To address this issue, the proposed method utilized a multi-scene cattle target segmentation approach based on the DeepLabV3+ model, which is known for its high performance in semantic image segmentation tasks, and it achieved exceptional results on various benchmarks, making it a reliable choice for accurate segmentation. Firstly, the backbone network of MobileNetV2 was selected due to its powerful feature extraction ability and robustness. Secondly, the Decoder stage adopted a layered feature fusion method and introduced SENet to strengthen feature fusion ability and focus the model on cattle target features, resulting in more detailed feature integration. Finally, the improved model was tested on a diverse and complex sample set, indicating accurate segmentation and the contour feature extraction of cattle targets with different breeds under challenging scenes. This approach exhibits robustness and strong applicability in the field of semantic segmentation, providing a promising solution for target segmentation and identification of cattle in precision livestock farming. It can also be applied to other field of image segmentation research.

The paper is organized as follows: Section 2 introduces the datasets used and the improved network model. Section 3 presents the experimental setup and performance evaluation methods. The segmentation results and model comparison experiments, as well as an application analysis, are described in Section 4, and conclusions are drawn in Section 5.

## 2. Materials and Methods

### 2.1. Data Acquisition Platform

In our experiments, the cattle dataset consisted of a cow dataset and beef dataset which were captured using a smartphone in field conditions at the Animal Husbandry Teaching Test Base, Northwest Agriculture and Forestry University, Yangling, China (see Li et al. [22]; https://github.com/MicaleLee/Database, accessed on 26 August 2022). The smartphone was fixed in a specific position to capture images of cattle with varying angles and scales, resulting in a relatively complex dataset.

**Figure 1:**
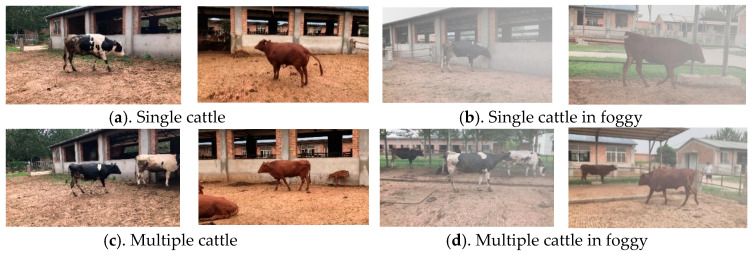
Sample dataset example.

Images were captured at a 1920 × 1080 resolution and saved in JPG format. Finally, a total of 781 images of dairy and beef cattle with multiple cattle, body area occlusion, and standing, walking, and lying postures in the natural breeding environment were selected to construct a complex and diverse dataset. Labelme software (https://link.zhihu.com/?target=https%3A//github.com/wkentaro/labelme, accessed on 26 August 2022) was used for annotation; the annotated images were saved in PNG format, and a sample from the dataset is illustrated in Figure 1. The image data of dairy and beef were divided into training and test sets in a ratio of 8:2. To further evaluate the performance of the proposed model, 50% of the images in the test set were randomly selected for fogging processing to simulate real scenarios (such as foggy weather) which may induce poor vision in outdoor environments. These rigorous measures were taken to ensure accurate evaluation of the proposed model’s performance on a complex and diverse dataset.

### 2.2. Improved DeepLabV3+ Segmentation Model

The DeepLabV3+ model [23], which includes an Encoder–Decoder structure composed of Input, Encoder, Decoder, and Output stages, is a popular semantic image segmentation tool. In the context of multi-scenario cattle contour extraction tasks, the original DeepLabV3+ model suffers from a few shortcomings, such as a large number of parameters, low segmentation accuracy, and poor generalization ability. Therefore, this study proposes an improved version of DeepLabV3+. The MobileNetV2 network was used as the backbone network of DeepLabV3+ to enhance the model’s ability to extract cattle contour features, reduce the amount of model parameters, and optimize model processing speed. Moreover, the original DeepLabV3+ model only performs four-fold down-sampling of the underlying feature layer, high-level feature layer fusion, and two rounds of four-fold up-sampling for decoding operations. This approach results in a considerable loss of spatial position information during the high-level and low-level feature fusion up-sampling process. Consequently, the actual segmentation effect on complex multi-scene datasets is poor. To address this limitation, we propose a novel layer-by-layer feature fusion strategy that combines U-Net network decoding methods. In the Decoder stage of DeepLabV3+, the low-level high-resolution features of three different scales extracted by the Backbone in the Encoder stage with high-level semantic feature information were fused. Then, the 3 × 3 convolution, BN, ReLU and 2× bilinear up-sampling modules were used to extract detailed features and restore the size of the feature map. After the last feature fusion, two 3 × 3 convolutional layers were stacked to greatly increase the nonlinear characteristics of the network without losing the resolution of the feature map, deepen the depth of the network to realize the cross-channel integration and interaction of information, and improve the feature segmentation performance of cattle targets.

**Figure 2:**
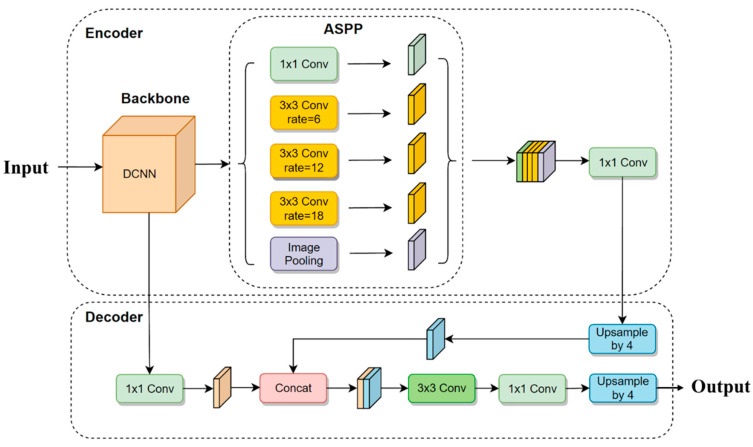
Network structure of DeepLabV3+.

**Figure 3:**
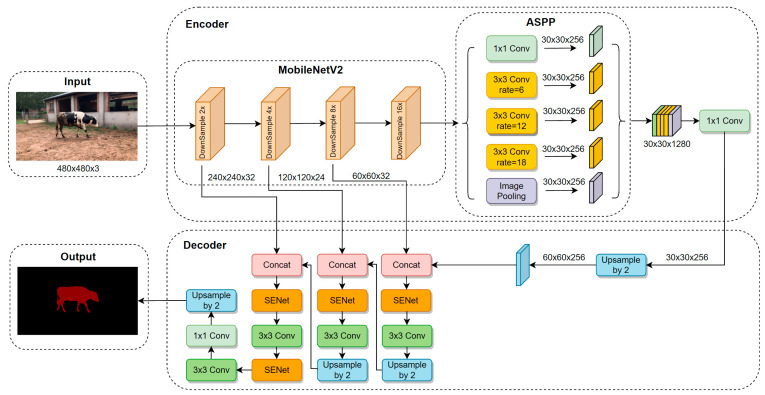
Network structure of Imp-DeepLabV3+.

The layer-by-layer feature fusion method employed in the proposed approach leads to shallow features in the up-sampled feature map, resulting in the redundancy of network information and the inability to highlight crucial feature information. Therefore, the Squeeze-and-Excitation Network (SENet) was introduced in the proposed method, which emphasizes the main features of cattle targets and suppresses unimportant features such as background by establishing the association between feature map channels and capturing a large amount of interaction information between channels. The resulting improved model is called Imp-DeepLabV3+. The original V3 framework is shown in Figure 2, and the improved V3 framework is shown in Figure 3.

In the Imp-DeepLabV3+ model, the backbone network and Decoder are improved. Specifically, the 2× bilinear up-sampling module, the 3-time channel information fusion module, and SENet retain the spatial information lost in multiple sampling and enhance the network’s attention to target characteristics of cattle. Consequently, the edge segmentation ability of cattle trunk contours in various scenes is significantly improved.

### 2.3. MobileNetV2 Network

**Figure 4:**
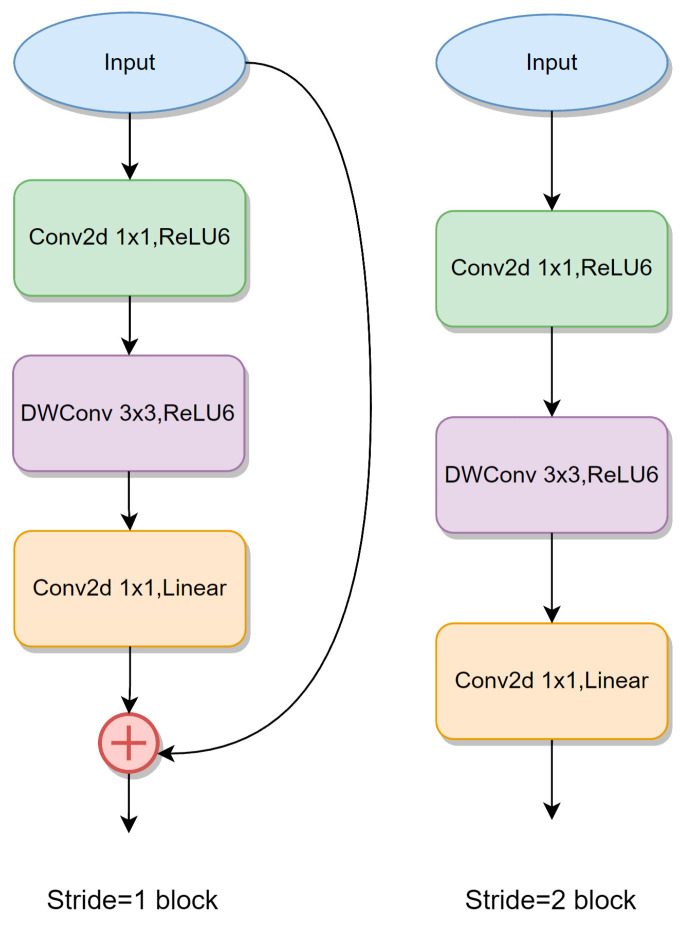
Inverted Residual and Linear Bottleneck structure of MobileNetV2.

MobileNetV2 [24] is an improved version of MobileNetV1 that uses the Inverted Residual and Linear Bottleneck structure. This architecture boasts several advantages, including low memory consumption, fast inference speed, and a strong feature extraction ability. Figure 4 illustrates the Bottleneck structure of MobileNetV2. To address the poor feature extraction ability of the Xception network of DeepLabV3+, the first four down-sampling feature extraction layers of MobileNetV2 were used as the backbone extraction network in the Encoder stage of DeepLabV3+ network. At the same time, atrous convolution and stride = 1 operation were used in the 16× down-sampling layer and 32× down-sampling layer of MobileNetV2 to increase the receptive field while maintaining the resolution of the feature map. The backbone network structure is shown in Table 1. 

### 2.4. Layer-by-Layer Feature Fusion of Decoder 

**Figure 5:**
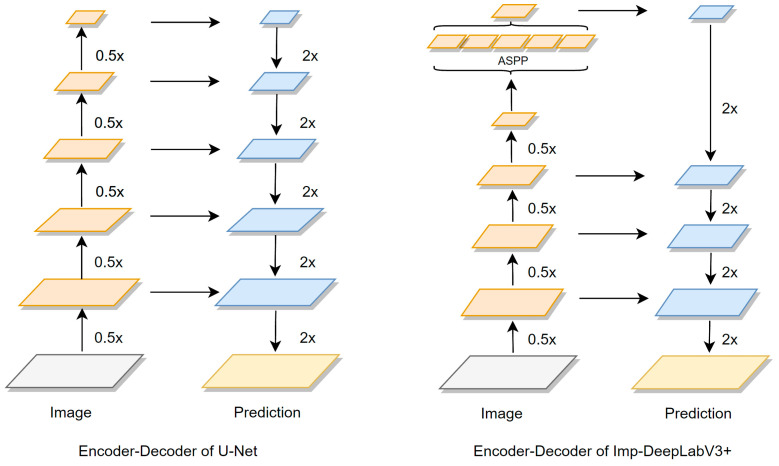
Encoder–Decoder network structure.

The DeeplabV3+ network’s Decoder component only employs two 4× bilinear up-sampling layers and a feature fusion layer, which can be insufficient for effectively connecting the multi-scale feature map and may result in the significant loss of spatial feature information with large up-sampling magnifications. As a consequence, the segmentation model’s performance on cattle targets may suffer. To address this challenge, we integrated the layer-by-layer feature fusion method used by U-Net in its Decoder stage with the MobileNetV2 backbone network. Specifically, the low-resolution, high-level feature map with semantic feature information obtained by performing deep feature extraction on cattle target images through the Encoder was fused layer by layer with the high-resolution, low-layer feature maps from the 8× down-sampling, 4× down-sampling, and 2× down-sampling layers. After feature fusion, 3 × 3 convolution, BN, ReLU, and 2× bilinear up-sampling layer were used to extract the detailed information of the fusion features and gradually restore the feature map size. Its Encoder–Decoder structure is shown in Figure 5. The improved Decoder adopts four 2× bilinear up-sampling and three feature fusion modules to achieve more detailed up-sampling operations, which greatly improves segmentation performance.

### 2.5. SENet

The Squeeze-and-Excitation Network (SENet) is an attention mechanism that focuses on the feature map channel dimension [25]. It facilitates feature selection and weights assignment by squeezing and then exciting the target features of cattle in each channel dimension. This mechanism assigns feature weights to the feature map through the Scale operation, enhancing the segmentation task network’s sensitivity to key features of cattle targets.

**Figure 6:**
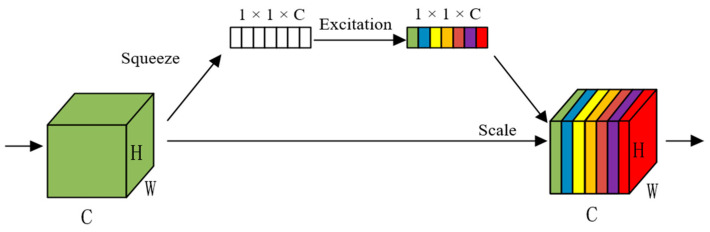
The structure of the Squeeze-and-Excitation Network.

Specifically, the method begins by inputting the feature map with a H × W × C resolution. The feature map is then pooled using the Squeeze operation over a global average, compressing the feature map to generate a feature vector of 1 × 1 × C. The Excitation operation subsequently establishes correlations between the channels of the cattle target feature map by utilizing two fully connected layers with H-Swish and H-Sigmoid nonlinear activation functions to generate corresponding weights for each channel. The Scale operation assigns weights to the feature map, emphasizing the crucial features of the cattle target while suppressing unimportant features such as background. SENet enables the model to focus its attention primarily on critical features of the cattle target. The SENet structure is shown in Figure 6.

## 3. Test Platform and Model Evaluation Indicators

### 3.1. Test Platform and Comparison Models

In this experiment, the model was built, trained, and verified by using the Pytorch1.12.1 deep learning framework with the Ubuntu20.04 operating system and NVIDIA RTX3080 graphics card. CUDA11.3 computing architecture was used, and CUDNN was added to the environment to accelerate computing power. In the process of the improved model test, the sample image resolution was 480 × 480, batch size was 4, and the initial learning rate was 0.01. The poly learning rate optimization method was adopted, and the initial momentum was 0.9. SGD was used as the optimizer, and all references models were trained according to this parameter for 60 epochs. Overall, these hyperparameter choices were made based on a comprehensive assessment of dataset complexity, model performance, and computational constraints. At the same time, in order to verify the performance of Imp-DeepLabV3+, it was compared and analyzed with a FCN [26], LR-ASPP [27], U-Net [28], and DeepLabV3+.

### 3.2. Evaluation Indicators

In order to comprehensively evaluate the performance of the improved model, common indicators of semantic segmentation were utilized. These indicators include Pixel Accuracy (*PA*), Class Pixel Accuracy (*CPA*), Mean Pixel Accuracy (*MPA*), Intersection over Union (*IoU*), and Mean Intersection over Union (*MIoU*). Pixel Accuracy (PA) represents the percentage of correctly predicted pixel values to the total number of pixel values. Class Pixel Accuracy (*CPA*) and Mean Pixel Accuracy (*MPA*) measure the model’s ability to partition pixels accurately between the background, cows, and beef. *IoU* and *MIoU* represent the ratio of the intersection and union between the true and predicted values, where *MIoU* is the average of the *IoU* values for each category. This metric reflects the degree of coincidence between the predicted segmentation region and the labeled region. The *PA*, *CPA*, *MPA*, *IoU*, and *MIoU* equations are shown in Equations (1)–(5), respectively.

(1) $$PA=\frac{TP+TN}{TP+FP+FN+TN}$$

(2) $$CPA=\frac{TP}{TP+FP}$$

(3) $$MPA=\frac{\sum CPA}{N_{C}}$$

(4) $$IoU=\frac{TP}{TP+FP+FN}$$

(5) $$MIoU=\frac{\sum IoU}{N_{C}}$$

where *TP* represents the number of positive sample pixels that the model is correctly segmented; *TN* represents the number of negative sample pixels that the model segmented correctly; *FN* represents the number of pixels that the model is incorrectly segmented into negative samples; *TN* represents the number of pixels in which the model incorrectly splits positive samples; *NC* is the number of subdivision categories (background, cows, and beef).

## 4. Results and Analysis

### 4.1. Segmentation Results of Different Models

To further evaluate the effectiveness of the proposed method, the improved Imp-DeepLabV3+ model was compared with several other semantic segmentation models, including FCN, LR-ASPP, U-Net, and DeepLabV3+. The results, as shown in Table 2, demonstrate that the Imp-DeepLabV3+ model achieved *PA*, *MPA*, and *MIoU* scores of 99.4%, 98.1%, and 96.8%, respectively, which are superior to the other models evaluated. Compared with FCN, LR-ASPP, U-Net, and DeepLabV3+, the Imp-DeepLabV3+ model achieved a higher *MPA* score by 1%, 3%, 29.1%, and 30.2%, respectively. Similarly, the *MIoU* score of the Imp-DeepLabV3+ model surpassed the scores of these models by 1.9%, 4.8%, 37.6%, and 34.8%, respectively.

Therefore, the results indicate that the proposed Imp-DeepLabV3+ model provides optimal segmentation performance in the task of multi-scene cattle target segmentation, surpassing the performance of other state-of-the-art models. These results show that the Imp-DeepLabV3+ model has significant potential for practical applications, such as animal husbandry management and monitoring.

**Figure 7:**
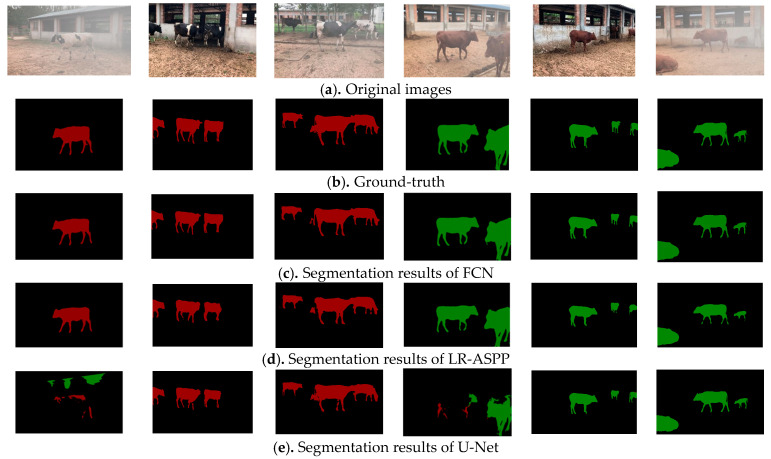

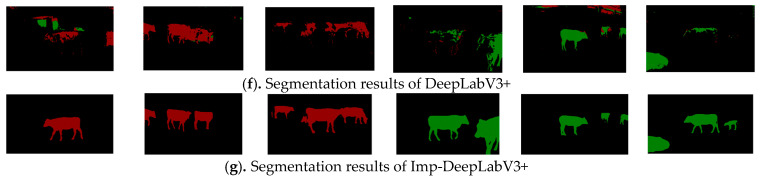
Segmentation results of different models for cattle targets.

The test set used in this study included a variety of sample images, such as single-cow, single-beef, multiple cow fogging, and multiple beef cattle fogging. The segmentation results of each model in a multi-scene complex environment are presented in Figure 7, where the cow segmentation color is red, and the beef cattle segmentation color is green.

From Figure 7, it can be seen that the proposed Imp-DeepLabV3+ model outperforms the comparison models in terms of single- and multiple-cattle target segmentation in natural environments, particularly so in the fogged sample images. The original DeepLabV3+ model exhibited a low segmentation accuracy for cattle targets in foggy conditions, with a large area of misidentification in the background area and failure to recognize cattle targets accurately. In addition, other comparison models are not finely segmented at the edges of cattle. In contrast, the improved Imp-DeepLabV3+ model accurately separated cattle targets from the background, which closely approximated the labeled image; the second-best model in this regard was the FCN.

Moreover, the proposed model incorporates the Squeeze-and-Excitation Network (SENet) in the Decoder stage to reweight the convolutional feature channels, which enhances the interdependence between key features of cattle targets and highlights the importance of cattle contour feature areas. This modification improves the feature extraction ability and segmentation performance of the model.

In summary, these results show that the Imp-DeepLabV3+ model is a robust and effective approach for cattle target segmentation in a wide range of complex environments. This study provides meaningful insights for animal husbandry management and monitoring.

### 4.2. Comparative Analysis of DeepLabV3+ Improved Model

The segmentation performance of different models, including Imp-DeepLabV3+, DeepLabV3+, M2-DeepLabV3+, and M2-U-DeepLabV3+, were compared and analyzed for a variety of cow and beef cattle datasets which included samples of many images taken in sunny and foggy conditions. The DeepLabV3+ model served as the original model, with Xception serving as the backbone network. M2-DeepLabV3+ utilized the MobileNetV2 network as the backbone, and M2-U-DeepLabV3+ employed a layer-by-layer feature fusion method in the Decoder stage. Finally, Imp-DeepLabV3+ incorporated the structure of SENet into the M2-U-DeepLabV3+ model to improve performance.

The experimental results in Table 3 prove that the M2-DeepLabV3+ model shows significant improvement in overall segmentation performance compared to the original model, indicating that the MobileNetV2 backbone network enhanced feature extraction ability, generalization ability, and segmentation effectiveness for the complex datasets. Furthermore, the M2-U-DeepLabV3+ model presented superior segmentation performance to the M2-DeepLabV3+ model, resulting in an increase in *MPA* and *MIoU* by 0.4% and 1.4%, respectively. Regarding the segmentation accuracy of cow and beef pixels, the Imp-DeepLabV3+ model with the SENet structure showed remarkable segmentation performance metrics, including a *PA* of 99.4%, *MPA* of 98.1%, *MIoU* of 96.8%, *CPA* (Cow) of 97.5%, CPA (Beef) of 97.0%, *IoU* (Cow) of 95.0%, and *IoU* (Beef) of 95.9%, which exceeds the original DeepLabV3+ model by 6.7%. Specifically, there was a 30.2% increase in *MPA* and a 34.8% increase in *MIoU*, indicating significant improvement in the Imp-DeepLabV3+ model’s segmentation performance.

### 4.3. Segmentation Effect under Different Datasets

To further evaluate the generalization ability of the Imp-DeepLabV3+ model, two additional test sets were constructed: one containing all sample data simulated by sunny simulated by and the other containing all sample data simulated by fog processing. The results are shown in Table 4 and Table 5. Our evaluation of the model’s performance on these two extreme datasets revealed that the Imp-DeepLabV3+ model outperformed the original model, especially with respect to the fogging test set. As shown in Table 5, the original model achieved only 19.5% and 17.2% in *CPA* (Cow) and *CPA* (Beef), respectively, and *IoU* (Cow) and *IoU* (Beef) were only 2.5% and 2.4%. In contrast, the Imp-DeepLabV3+ model displayed superior segmentation performance with a *CPA* (Cow) of 96.7%, *CPA* (Beef) of 95.4%, *IoU* (Cow) of 94.4%, and *IoU* (Beef) of 94.9%, which represents a 77.2% and 92.9% increase in *CPA* (Cow) and *CPA* (Beef), respectively, compared to the DeepLabV3+ model. *IoU* (Cow) and *IoU* (Beef) also increased by 77.2% and 92.5%, respectively. 

Overall, the Imp-DeepLabV3+ model demonstrated strong robustness and achieved accurate cattle target segmentation in multi-scene complex environments. Furthermore, the model exhibited good segmentation results on the original test set and under the sunny, foggy, or combined test sets, thereby validating its suitability for practical applications.

### 4.4. Application Analysis

The feeding environment plays a crucial role in the growth and development of animals. Effectively and accurately extracting cattle targets and analyzing their relationship with environmental factors is one of the most effective ways to control the breeding environment. In this context, image segmentation is an important part of analyzing cattle behavior characteristics based on computer vision technology. Segmenting cattle targets from images provides easy-to-analyze and easily understandable image representation for cattle behavior feature extraction, image analysis, pattern recognition, and other applications. Therefore, on the basis of obtaining the target area, the tracking method can be further studied, which is expected to achieve the continuous tracking and monitoring of video-based targets, such as the length of time the target stays in a certain area, the frequency of certain behaviors, etc. This is beneficial for further analyzing animal habits. 

**Figure 8:**
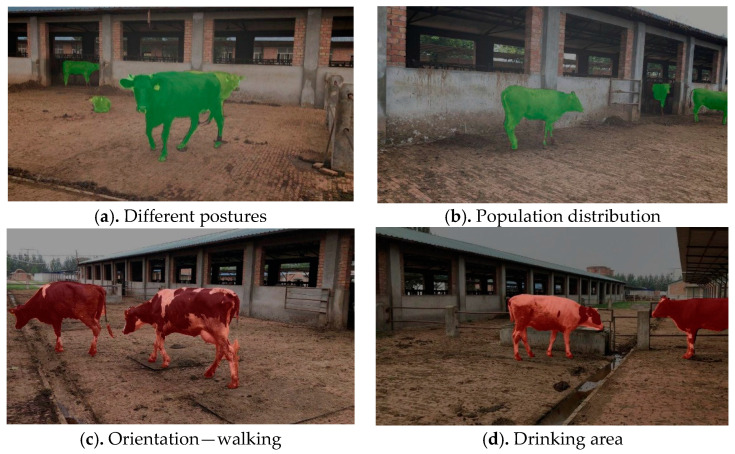
Original image segmentation display.

As presented in Figure 8, once the target area is segmented, the posture, orientation, and interaction information of the cattle in the scene can be further analyzed. For example, according to the contour information (length, width, perimeter, curvature, etc.) of the segmented target, the mapping relationship between the contour information and the animal posture can constructed, meaning that the posture recognition of cattle (e.g., standing, lying down, and turning) can be evaluated (Figure 8a,b). According to the contour information, the orientation information of a cow can be obtained; for example, there is a large difference between the head contour and the trunk contour of the cow, and the head area can be further obtained according to the difference. In addition, combined with the scene information, the orientation information of the head in the image can be further determined. In the monitoring video, real-time object detection can further analyze whether the cattle’s forward direction will exhibited scene interaction behavior, such as going to the drinking area, eating area, etc. (Figure 8c,d). 

### 4.5. Special Case Analysis

**Figure 9:**
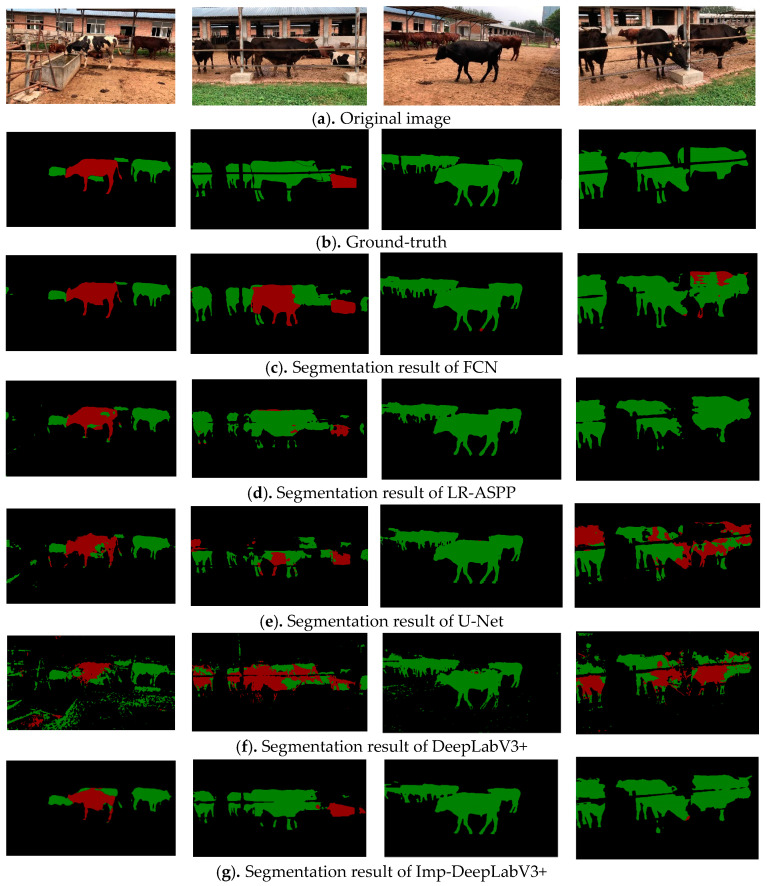
Segmentation results of different models in special cases.

To further verify the generalization and effectiveness of the proposed method, a comparative analysis was performed on the images of crowded cattle, railing occlusion, and mixed varieties. The results are shown in Figure 9. When the body hair of beef is dark, the beef will be mistakenly identified and divided into cows, that is, the green area is incorrectly marked as red. In particular, the U-Net and DeepLabV3+ comparison method yielded poor segmentation results, followed by FCN. The method proposed in this paper is superior to the comparison method. In addition, railing occlusion affects the integrity of target area segmentation, but since railings are regular objects, this problem can be improved by regular area filling. However, the phenomenon of missing detection exists when the distant small target area is segmented in the scene. Therefore, small-target segmentation performance should be further strengthened to improve the performance of the proposed model in different scenes.

### 4.6. Generalized Application Analysis of the Model

**Figure 10:**
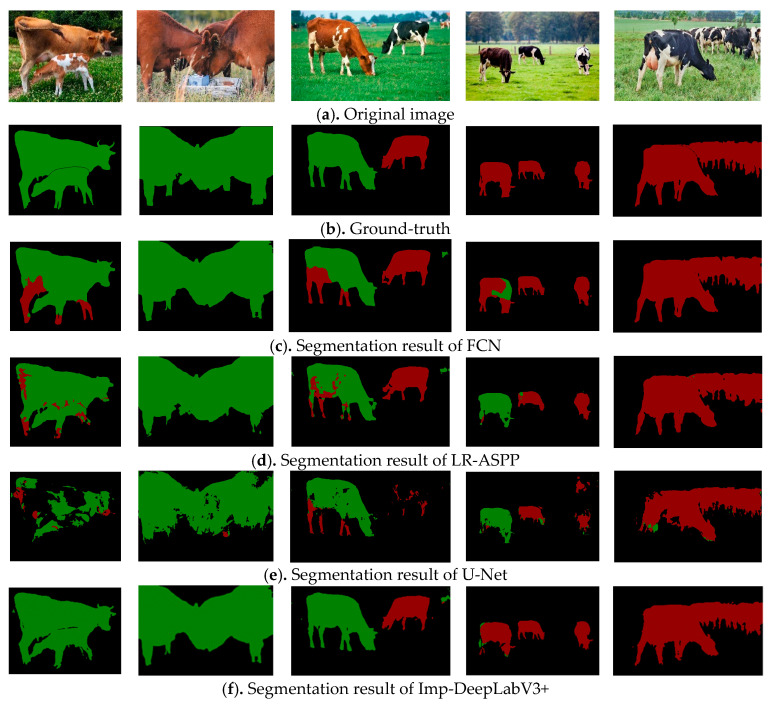
Segmentation results of different models using open-source cattle images.

In order to further verify the generalization and application of the proposed method, images of cattle in different open-source scenes were collected from the internet and used as test sets for verification, as shown in Figure 10. It can be seen that the segmentation effect of LR-ASPP and U-Net is poor; there was a serious loss of target area, and beef cattle and dairy cows were misidentified. The segmentation results of the FCN model showed improvement, but there were also cases of misidentification regarding beef cattle and dairy cows, and the proposed method demonstrated improvement in this aspect. In addition, the overall segmentation effect of Imp-DeepLabV3+ is also better than that of the comparison algorithm. Therefore, the model has good generalization and application. In other words, after the model is built, its stability and accuracy in different scenes are better than the comparison method. Moreover, the model can also be applied to other target segmentation studies.

## 5. Conclusions

In this study, an improved DeepLabV3+ semantic segmentation model was proposed for cattle target segmentation. To enhance the feature extraction and generalization capabilities of the segmentation network, MobileNetV2 was introduced, replacing the Xception network as the backbone network. In the Decoder stage, a layer-by-layer feature fusion method was adopted, and four 2× bilinear up-sampling modules, three feature fusion modules, and SENet structure were employed. This enables the effective decoding of spatial feature information, enabling the model to focus on important cattle targets features and the scene’s background while ignoring other unimportant features. Overall, the performance of the improved model in the task of cattle segmentation significantly improved. To further test the robustness of the proposed model, image samples that simulate the poor level of visibility one experiences on foggy days were added to the dataset, and we constructed a multi-scene and complex cattle target dataset. After using this dataset to verify the model and analyzing the results, it was found that the improved Imp-DeepLabV3+ model achieved a pixel accuracy (*PA*) of 99.4%, a mean pixel accuracy (*MPA*) of 98.1%, and a mean intersection over union (*MIoU*) of 96.8%, demonstrating outstanding segmentation performance. In future work, the mapping relationship between contour information and animal posture and behavior will be studied further, and the scene information will be combined to realize animal behavior recognition and behavior analysis in the scene.

The developed image segmentation algorithm exhibits high precision and strong robustness, but there are still some limitations, especially regarding large-area occlusion and congestion, which lead to a large loss of target information and cause the semantic segmentation model to experience difficulty when trying to accurately segment it. This is also a difficult problem in practical applications.

## Figures and Tables

**Table 1:** The backbone network structure of MobileNetV2.

Input	Operator	t	c	n	s	Rate
480 × 480 × 3	Conv2d	-	32	1	2	1
240 × 240 × 32	Bottleneck	1	16	1	1	1
240 × 240 × 16	Bottleneck	6	24	2	2	1
120 × 120 × 24	Bottleneck	6	32	3	2	1
60 × 60 × 32	Bottleneck	6	64	4	2	1
30 × 30 × 64	Bottleneck	6	96	3	1	1
30 × 30 × 96	Bottleneck	6	160	3	1	1
30 × 30 × 160	Bottleneck	6	320	1	1	2

Note: Input indicates the size of the input feature graph; Operator indicates the convolutional module operations performed by the layer network; t indicates Expansion factor in Bottleneck; c represents the number of output channels; n indicates the number of repetitions of the operation; s indicates step distance; and rate indicates the empty convolutional expansion rate.

**Table 2:** Segmentation results of different models (%).

Model	*PA*	*MPA*	*MIoU*	*CPA* (Cow)	*CPA* (Beef)	*IoU* (Cow)	*IoU* (Beef)
FCN	99.4	97.1	94.9	95.7	95.9	92.5	93.0
LR-ASPP	99.0	95.1	92.0	94.2	91.6	88.6	88.3
U-Net	94.2	69.0	59.2	48.3	61.1	44.6	38.7
DeepLabV3+	92.7	67.9	62.0	55.0	50.1	47.1	46.4
Imp-DeepLabV3+	99.4	98.1	96.8	97.5	97.0	95.0	95.9

**Table 3:** Ablation experiments under sunny and foggy images (%).

Model	*PA*	*MPA*	*MIoU*	*CPA* (Cow)	*CPA* (Beef)	*IoU* (Cow)	*IoU* (Beef)
DeepLabV3+	92.7	67.9	62.0	55.0	50.1	47.1	46.4
M2-DeepLabV3+	99.1	97.5	95.2	96.5	96.4	92.3	94.4
M2-U-DeepLabV3+	99.4	97.9	96.6	97.0	97.0	94.6	95.8
Imp-DeepLabV3+	99.4	98.1	96.8	97.5	97.0	95.0	95.9

**Table 4:** Ablation experiments of sunny test set (%).

Model	*PA*	*MPA*	*MIoU*	*CPA* (Cow)	*CPA* (Beef)	*IoU* (Cow)	*IoU* (Beef)
DeepLabV3+	97.2	93.7	85.9	89.3	93.7	75.6	84.9
M2-DeepLabV3+	99.3	98.3	96.1	97.3	98.0	93.5	95.5
M2-U-DeepLabV3+	99.4	98.5	97.0	97.6	98.3	95.1	96.5
Imp-DeepLabV3+	99.5	98.8	97.3	98.3	98.4	95.5	96.9

**Table 5:** Ablation experiments of foggy test set (%).

Model	*PA*	*MPA*	*MIoU*	*CPA* (Cow)	*CPA* (Beef)	*IoU* (Cow)	*IoU* (Beef)
DeepLabV3+	88.1	40.5	36.1	19.5	2.5	17.2	2.4
M2-DeepLabV3+	98.9	96.4	94.1	95.3	94.5	90.7	92.8
M2-U-DeepLabV3+	99.2	97.1	95.9	96.0	95.5	93.8	94.8
Imp-DeepLabV3+	99.3	97.3	96.2	96.7	95.4	94.4	94.9

## Data Availability

The data that support the findings of this study are available from corresponding author but restrictions apply to the availability of these data, which were used under license for the current study, and so are not publicly available. Data are however available from the authors upon reasonable request and with permission of the corresponding author.
